# Desmosterolosis: an illustration of diagnostic ambiguity of cholesterol synthesis disorders

**DOI:** 10.1186/1750-1172-9-94

**Published:** 2014-06-25

**Authors:** Cristina Dias, Rosemarie Rupps, Benjamin Millar, Kunho Choi, Marco Marra, Michelle Demos, Lisa E Kratz, Cornelius F Boerkoel

**Affiliations:** 1 Department of Medical Genetics, University of British Columbia, 4500 Oak St., Vancouver, British Columbia, V6H 3N1, Canada; 2 Child and Family Research Institute, Children’s and Women’s Health Centre of British Columbia, 950 West 28th Ave., Vancouver, British Columbia, V5Z 4H4, Canada; 3 Canada’s Michael Smith Genome Sciences Centre, 570 W 7th Ave, #100, Vancouver, British Columbia, V5Z 4S6, Canada; 4 Division of Pediatric Neurology, Department of Pediatrics, University of British Columbia, Vancouver, 4480 Oak Street, Vancouver, British Columbia, V6H 3V4, Canada; 5 Department of Neurogenetics, Kennedy Krieger Institute, 707 N. Broadway, Baltimore, Maryland MD 21205, USA; 6 Wellcome Trust Sanger Institute, Hinxton, Cambridge, UK

**Keywords:** DHCR24, Desmosterol, Intellectual disability, Cholesterol biosynthesis, Exome sequencing

## Abstract

Desmosterolosis is an autosomal recessive disorder of cholesterol biosynthesis caused by biallelic mutations of *DHCR24* (homozygous or compound heterozygous), which encodes 3-β-hydroxysterol Δ-24-reductase. We report two sisters homozygous for the 571G>A (E191K) *DHCR24* mutation. Comparison of the propositae to other reported individuals shows that psychomotor developmental delay, failure to thrive, dysgenesis of the corpus callosum, cerebral white matter atrophy and spasticity likely constitute the minimal desmosterolosis phenotype. The nonspecific features of desmosterolosis make it difficult to suspect clinically and therefore screening for it should be entertained early in the diagnostic evaluation.

## Findings

### Background

Desmosterolosis is an infrequently reported disorder of cholesterol biosynthesis causing syndromic intellectual disability (ID) arising from biallelic mutations (homozygous or compound heterozygous) in *DHCR24. DHCR24* encodes 3-β-hydroxysterol Δ-24-reductase (DHCR24) [1,2], which catalyzes the C-24 NADPH-dependent reduction of the 24–25 double bond of cholesterol precursors [3,4].

### Case report

We present two sisters with syndromic ID and desmosterolosis. Following uncomplicated pregnancies, they were born at term with normal growth parameters. Each had transient neonatal seizures. Their family history was noncontributory.

Beginning in infancy, they manifested growth restriction and delayed milestones for speech, fine and gross motor, and adaptive development. Patient 1 was able to walk with support and communicate with short phrases by 6 years; Patient 2 developed these skills by 8 years. Patient 1 had an IQ of 42 at 10.5 years. Patient 2 had an IQ of 46 at 5.5 years. Neither lost skills although Patient 1 had progressive sensorineural hearing loss.

**Figure 1:**
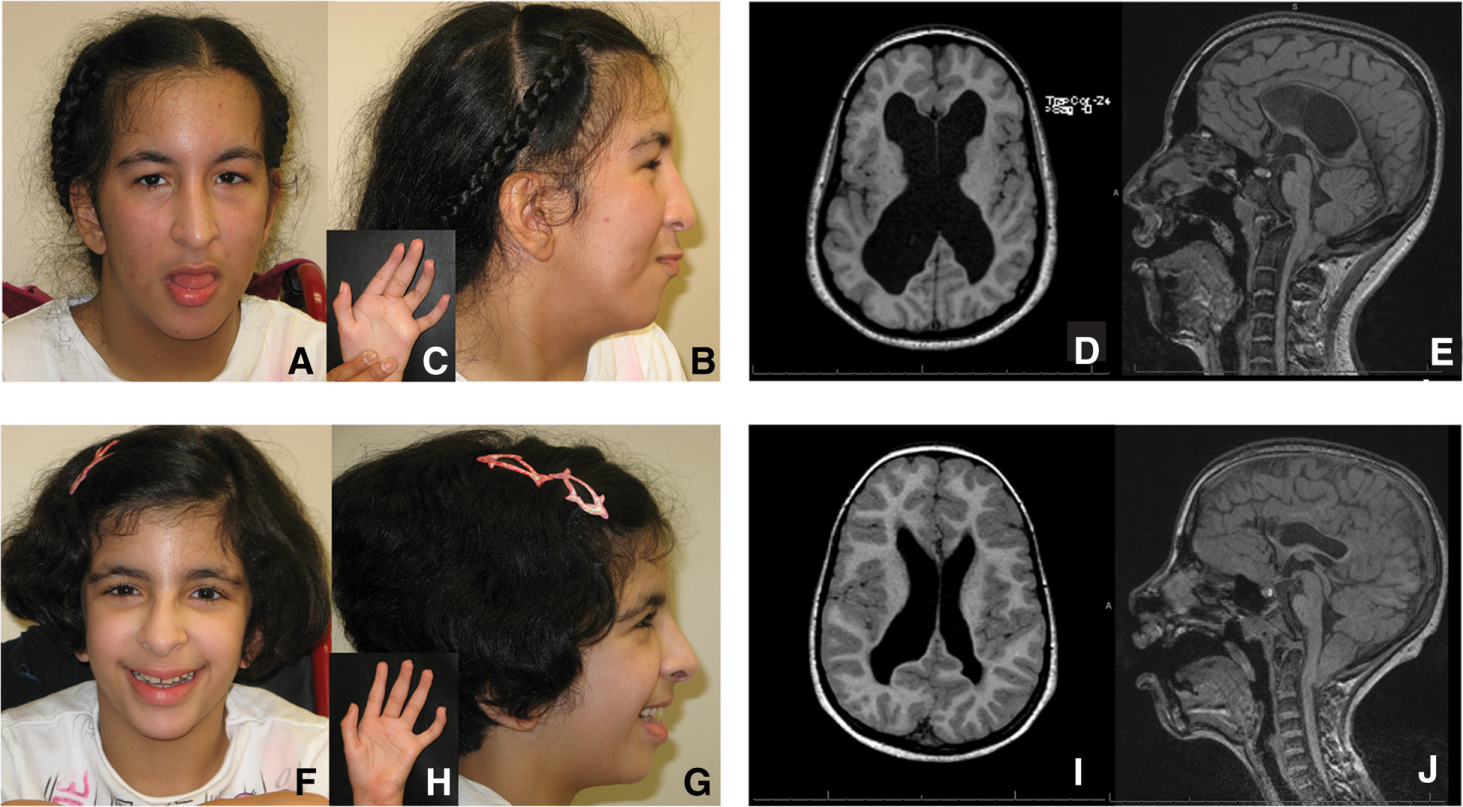
**Clinical and MRI features of siblings with Desmosterolosis. A** to **C**: Patient 1 craniofacial and hand features at age 14.8 years. Her dysmorphic features show scaphocephaly, tall forehead with bitemporal narrowing, short palpebral fissures, long nose, hypoplastic nasal alae, prominent columella, and low-set posteriorly rotated ears. **D**, **E** (Axial T1, Sagittal FLAIR): Patient 1 brain MRI showing white matter volume loss, dilated ventricles, thin corpus callosum, and peg-like cerebellar tonsils displaced into the upper cervical canal through the foramen magnum (Chiari I malformation). **F** to **H**: Patient 2 craniofacial and hand features at age 10.1 years, similar to Patient 1. **I**, **J** (Axial T1 and Sagittal FLAIR): Patient 2 brain MRI showing prominent and irregular ventricles, thin corpus callosum, and Chiari I malformation.

On examination at 13.8 and 9.1 years, respectively, each had similar dysmorphic features. Patient 1 had short stature (<1^st^ centile) with proportionate limbs, a weight of 41 kg (13^th^ centile), and a head circumference of 55.2 cm (86^th^ centile). Patient 2 had a height of 117.8 cm (1^st^ centile), a weight of 21.3 kg (3^rd^ centile), and a head circumference of 49.5 cm (4^th^ centile). They had mild generalized hirsutism, facial dysmorphism, high arched hard palates, optic atrophy, nystagmus, spasticity, deep tendon hyperreflexia, camptodactyly of the fingers and toes, and muscle wasting, particularly of the thenar and hypothenar muscles (Figure 1A, B, C, F, G and H). Patient 1 also had a midline cleft of the soft palate as well as a short neck and large ears (>97^th^ centile). Patient 2 also had myopia.

### Investigations

Each had extensive non-diagnostic laboratory testing. This included normal profiles for urine organic acids, urine purines and pyrimidines, plasma amino acids, and plasma very long chain fatty acids as well as urine mucopolysaccharide and oligosaccharide screens, liver function studies, transferrin isoelectric focusing and levels for lactate, ammonia, uric acid, albumin, creatinine phosphokinase, and thyroid stimulating hormone. Each had a normal karyotype and no evidence of a genomic deletion or duplication detectable by array comparative genomic hybridization. Patient 1 also had normal nucleotide excision repair assays, electromyography and nerve conduction studies. Patient 2 had normal complete blood counts and levels for copper, ceruloplasmin and blood acylcarnitines; her EEG identified no focal seizure activity. The patients were unavailable for additional testing, specifically plasma sterols, which were not performed on initial assessment.

Radiological assessment showed that both had dislocated radial heads and bilateral equinovarus. In addition, Patient 1 had a small and deformed pelvis, lumbar scoliosis, and moderate osteopenia. Patient 2 had parietal foramina.Magnetic resonance imaging (MRI) identified mild brain atrophy, asymmetric ventriculomegaly, a thin corpus callosum, and a Chiari I malformation (Figure 1D, E, I and J) In addition, Patient 1 had a sacral cyst suggestive of a meningocele or dural/perineural cyst.

### Exome sequencing and biochemical confirmation of desmosterolosis

**Figure 2:**
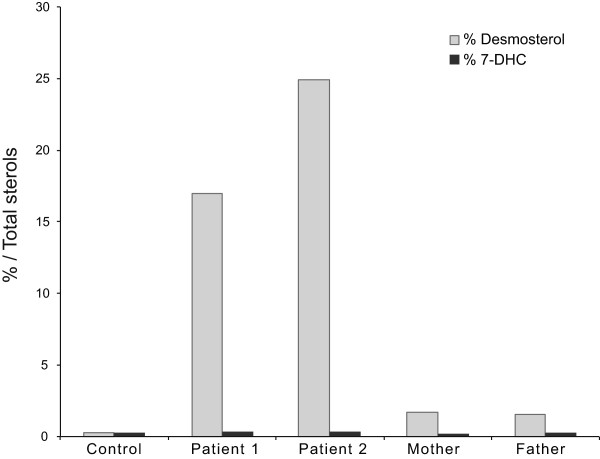
**Biochemical confirmation of desmosterolosis.** Comparative sterol profiles for the patients (second and third bar sets), heterozygous parents (fourth and fifth bar sets), and unaffected control (first bar set). Sterols were measured in lysates from lymphoblasts cultured in delipidated medium for 3 days and showed a 17–25 fold increased ratio of desmosterol to total sterols in comparison to controls, whereas as heterozygous parents present a 1.7 and 1.5 increase, respectively. Each bar represents the average of 3 technical replicates. 7-DHC is represented as an internal control.

Exome sequencing (for methods see Additional file 1) [5] identified a homozygous *DHCR24* mutation (NM_014762.3:c.571G>A; p.E191K), a recognized cause of desmosterolosis (Additional file 1, Supplementary methods and Additional file 2: Figure S1A) [2]. Sanger sequencing confirmed this and the carrier state of the parents (Additional file 2: Figure S1B). As predicted, gas chromatography-mass spectroscopy analysis of lysates from cultured lymphoblastoid cells [6] from the propositae detected an increased ratio of desmosterol to total sterols (Figure 2). All other sterols measured (Supplementary methods) including cholesterol and 7-dehydrocholesterol were within normal range compared to healthy controls.

## Conclusions

We present two sisters with the biallelic mutation NM_014762.3:c.571G>A in *DHCR24*. The recurrence of this mutation [2] in a different ethnic group implies that this mutation arose independently and suggests that mutations altering only certain amino acids give rise to a viable human with desmosterolosis (Table 1).

**Table 1:** Clinical features of reported patients with Desmosterolosis

**Feature**	**Present family**		**Previously reported cases of desmosterolosis**				**Frequency (n = 9)**
	**Patient 1**	**Patient 2**	**Patient 3**^**1**^	**Patients 4**-**7**^**2**^	**Patient 8**^**3**^	**Patient 9**^**4**^	
Mutation	c.[571G>A] + [571G>A] p.[E191K] + [E191K]		c.[571G>A] + [571G>A] p.[E191K] + [E191K]	c.[307C>T] + [307C>T] p.[R103C] + [R103C]	c.[281G>A] + [1438G>A] p.[R94H] + [E480K]	c.[1412A>C] + [881A > C;918G>C] p.[Y471S] + [N294T; K306N]	
Protein domain	FAD-binding domain		FAD-binding domain	FAD-binding domain	FAD-binding domain + C terminal cytoplasmic domain	C terminal cytoplasmic domain	
Ancestry	Middle Eastern		European	Israeli Bedouin		European	
Failure to thrive	1	1	1	4/4	1	n.a.	8/8
Short stature	1	1	1	n.a.	n.a.	0	3/4
Microcephaly	0	0	1	4/4	0	0	5/9
Macrocephaly	0	0	0	0/4	1	1	2/9
Microretrognatia	1	0	1	4/4	1	1	8/9
Cleft palate	1	0	1	0	0	1	3/9
Facial features	Dolicocephaly; bitemporal narrowing; low set ears; short downslanting PF; prominent columella; cleft palate	Dolicocephaly; bitemporal narrowing; low set ears; short downslanting PF; prominent columella;	Downslanting PF; bilateral epicanthal folds		Prominent forehead; Short nose; anteverted nares; telecanthus;	Frontal bossing; hypoplastic nose; low set ears; cleft palate	
ID/DD	1	1	1	4/4	1	n.a.	8/8
Spasticity	1	1	1	4/4	n.a.	n.a.	7/7
Distal arthrogryposis	1	1	1	4/4	1	0	8/9
Large joint contractures	0	1 (talipes)	1 (talipes)	n.a.	1	1	4/5
Shortening of the limbs	0	0	0	n.a.	1	1	2/5
ACC (partial/full)	1	1	1	4/4	1	1	9/9
Ventriculomegaly	1	1	0	4/4	1	1	8/9
Cerebral WM atrophy	1	1	1	4/4	1	n.a.	8/8
Cerebellar WM atrophy	1	1	n.a.	2/2	n.a.	n.a.	4/4
Nystagmus /strabismus	1	1	0	3/4	0	n.a.	5/8
Seizures	1	1	0	3/4	n.a.	n.a.	5/7
Other features	SNHL; Hirsutism	Parietal foramina. Hirsutism	Cutis aplasia; Limb anomalies; PDA;		Hydrocephalus	Osteosclerosis; ambiguous genitalia; anomalous pulmonary venous drainage; renal hypoplasia; death at 1 h	
Functional assays					Expressed both mutations in c.cerevisiae (separately) with significant ⇩enzyme activity	Expressed mutations in c.cerevisiae with significant ⇩enzyme activity (including compound het)	

References: 1: Waterham et al. [2], Andersson et al. [8]; 2: Zolotushko et al. [7]; 3: Schaaf et al. [9]; 4: FitzPatrick et al. [1], Waterham et al. [2].

*Abbreviations*: *ACC* agenesis of the corpus callosum, *DD* developmental delay, *ID* intellectual disability, *n.a.* not available, *PF* palpebral fissures, *PDA* patent ductus arteriosus, *SNHL* sensorineural hearing loss, *WM* white matter, *h* hour, *het* heterozygote.

Assessing genotype-phenotype correlation, the propositae were discordant for microretrognathia, cleft palate, large joint contractures, deafness, and skull foramina (Table 1). In contrast, the previously reported four cousins of a consanguineous family were discordant for oculomotor abnormalities and seizures (Patients 4–7, Table 1). This might suggest either that the genetic backgrounds of the propositae are significantly different or that because of their consanguinity, the four family members reported by Zolotushko et al. [7] share other genomic or epigenetic variants modifying the expressivity of desmosterolosis.

Comparison of the propositae to Patient 3 (Table 1), who has the same *DHCR24* mutation, provides an assessment of interfamilial genotype-phenotype concordance [2]. They were discordant for dysmorphic facial features, oculomotor abnormalities, seizures, brain ventriculomegaly, cutis aplasia, limb anomalies, and congenital heart defects. They were concordant for ID, failure to thrive, short stature, spasticity, distal arthrogryposis, dysgenesis of the corpus callosum, and cerebral white matter atrophy. Comparison of the propositae to all reported in individuals with desmosterolosis (Table 1) [1,2,7-9] identifies ID, failure to thrive, spasticity, dysgenesis of the corpus callosum, and cerebral white matter atrophy as the minimal clinical phenotype for desmosterolosis. Distal arthrogryposis occurred in 8 of 9 individuals (Table 1). This minimal phenotype, which is not distinctive and the absence of sterol testing, explains the decade-long diagnostic odyssey of the propositae.

Review of all reported patients suggests a minimal genotype-phenotype correlation for desmosterolosis. Only individuals with mutations affecting the 3-β-hydroxysterol Δ-24-reductase cytoplasmic domain had rhizomelia (Patients 8 and 9, Table 1). Sharing this feature with the knockout mice [10], might suggest that mutations in the cytoplasmic domain disrupt enzyme function more severely than mutations in the FAD-binding domain (protein features of UniProt Q15392 [11]).

The neurological features of desmosterolosis might arise from either deficiency of cholesterol biosynthesis or the toxic effects of sterols accumulating upstream of 3-β-hydroxysterol Δ-24-reductase. Both mechanisms contribute to other disorders of cholesterol biosynthesis and thus likely apply here [12]. The non-progressive neuropathology in desmosterolosis is in keeping with the primary impact occurring during brain development.

In summary, the pleiotropy and nonspecificity of desmosterolosis explain the long diagnostic odyssey of the propositae. Also, findings of developmental delay, CNS malformation, spasticity (with or without distal arthrogryposis), short stature with and without limb anomalies are sufficient indication to screen for disorders of cholesterol biosynthesis.

### Patient consent and ethics approval

Individuals enrolled in the study gave informed consent for protocol H07-02142 (Vancouver, BC, Canada), approved by the University of British Columbia Research Ethics Board. Written informed consent was provided for the collection of samples, subsequent analysis and use of photographs by the parents of the children.

## Abbreviations

bp: base pair; CNS: Central nervous system; IQ: Intelligence quotient; DHCR24: 3-β-hydroxysterol Δ-24-reductase; EBP: Emopamil binding protein; FISH: Fluorescence in situ hybridization; ID: Intellectual disability; MRI: Magnetic resonance imaging; 7-DHC: 7-dehidrocholesterol; PE: Paired-end.

## Competing interests

The authors declare that they have no competing interests.

## Authors’ contributions

CD, RR and CFB interpreted the data and drafted and revised the manuscript. CD, BM, KC and LEK performed experimental work and interpreted the data. CFB, MD and RR provided clinical data. CD and MM performed and interpreted the bioinformatic analysis. All authors read and approved the final manuscript.

## Supplementary Material

Additional file 1 **Supplementary methods.** Exome sequencing and Gas chromatography-mass spectroscopy analysis. Click here for file

Additional file 2: Figure S1 Molecular confirmation of Desmosterolosis through exome sequencing and Sanger sequencing. NextGene (Softgenetics, Pennsylvania) view of the exome sequencing reads for one affected child (A) and dideoxy nucleotide sequencing validation (B) for the mother, father, and both affected children. The mother and father were heterozygous for NM_014762.3:c.571G > A (p.E191K) mutation. The propositae have the mutation in homozygosity. Click here for file
