# Supplementary material for: Desmosterolosis: an illustration of diagnostic ambiguity of cholesterol synthesis disorders
Source: Orphanet J Rare Dis. 2014 Jun 25;9:94. doi: 10.1186/1750-1172-9-94 (PMC4076431; doi:10.1186/1750-1172-9-94)
Supplement: Additional file 1 — Supplementary methods. Exome sequencing and Gas chromatography-mass spectroscopy analysis. [file 1750-1172-9-94-S1.pdf]

Additional file 1.
Supplementary methods (.pdf).

Exome sequencing and Gas chromatography-mass spectroscopy analysis

Exome sequencing was performed as previously described (Dias et al., 2012). Briefly, exome enrichment was performed using the Human All Exon kit SureSelect Target Enrichment System (Agilent Technologies, Santa Clara, CA). Flow-cell preparation and 76 to 100-bp (base pair) paired-end (PE) read sequencing were performed per manufacturer's protocol for the Illumina Genome AnalyzerIIx (Illumina, San Diego, CA). The short-read PE sequences were aligned to the human genome reference sequence (NCBI build 36; hg18) and sequence variants were called using the NextGENe software (Softgenetics, Pennsylvania).
Gas chromatography-mass spectroscopy analysis of cholesterol and its precursors in cultured lymphoblastoid cells from the propositae was performed. Briefly, $2x10^6$ lymphoblastoid cells were cultured in 9 mL of delipidated media (RPMI with 10% delipidated FBS) for 3 days and sterol levels were measured in cell lysates as previously described (Kelley, 1995). Three technical replicates were performed for each sample.

Cell lysates from the propositae showed a 17-25 fold increased ratio of desmosterol to total sterols (Fig.2C). Total sterols included: Cholesterol; 8-Dehydrocholesterol; Cholesta-8(9)-en-3ß-ol; 7-Dehydrocholesterol; Desmosterol; Lathosterol; 4α-Methyl-cholesta-8-en-3ß-ol; 4α-methylcholesta-7-en-3ß-ol, ; 4-Methylcholesta-8(9),24-dien-3ß-ol; 4,4-dimethylcholesta-8-en-3ß-ol; 4,4-dimethylcholesta-8(9),24-dien-3ß-ol; Dihydrolanosterol; and Lanosterol.
