# Supplementary material for: Desmosterolosis: an illustration of diagnostic ambiguity of cholesterol synthesis disorders
Source: Orphanet J Rare Dis. 2014 Jun 25;9:94. doi: 10.1186/1750-1172-9-94 (PMC4076431; doi:10.1186/1750-1172-9-94)

Additional file 2. **Dias et al. OJRD 2014**

Supplementary figure (.pdf).

Molecular confirmation of Desmosterolosis through exome sequencing and Sanger sequencing

NextGene (Softgenetics, Pennsylvania) view of the exome sequencing reads for one affected child (A) and dideoxy nucleotide sequencing validation (B) for the mother, father, and both affected children. The mother and father were heterozygous for NM_014762.3:c.571G>A (p.E191K) mutation. The propositae have the mutation in homozygosity.

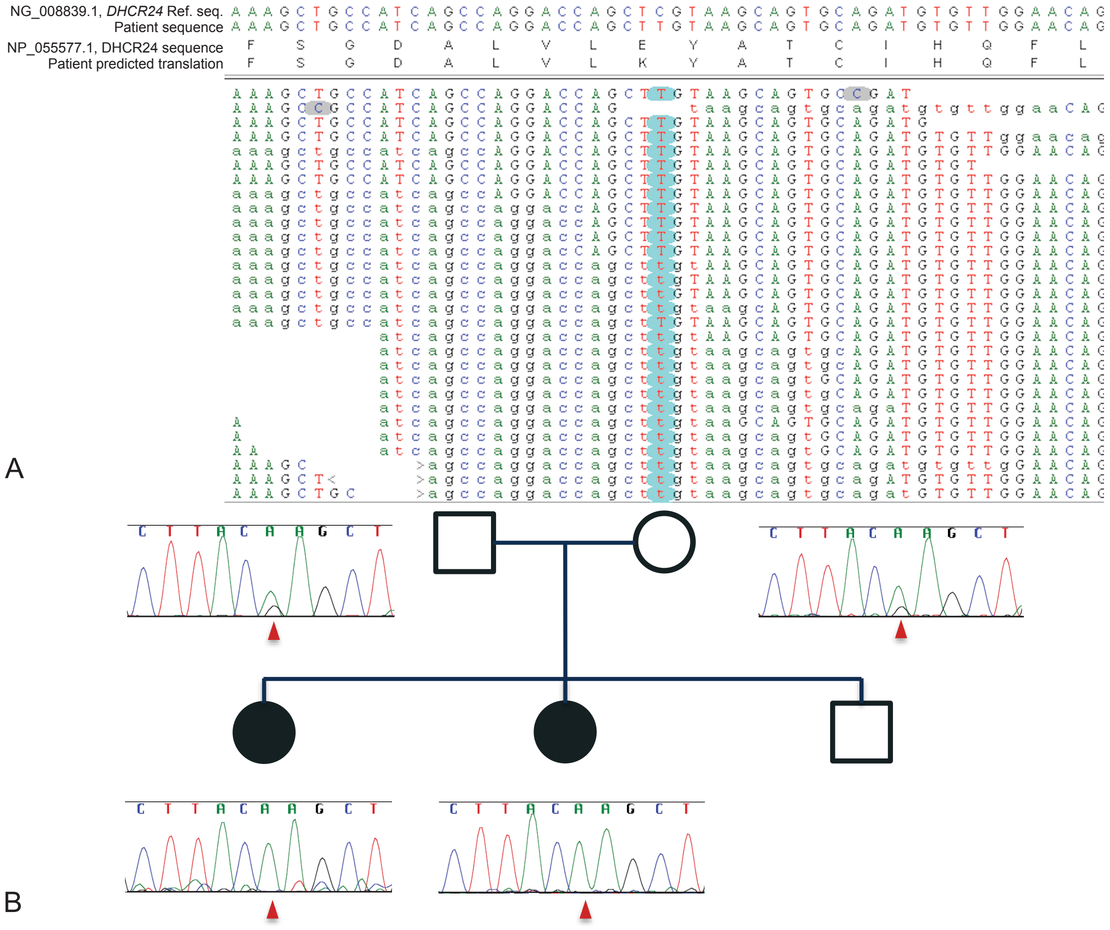

Supplement: Additional file 2: Figure S1 — Molecular confirmation of Desmosterolosis through exome sequencing and Sanger sequencing. NextGene (Softgenetics, Pennsylvania) view of the exome sequencing reads for one affected child (A) and dideoxy nucleotide sequencing validation (B) for the mother, father, and both affected children. The mother and father were heterozygous for NM_014762.3:c.571G > A (p.E191K) mutation. The propositae have the mutation in homozygosity. [file 1750-1172-9-94-S2.pdf]
